# Immunohistochemical Evaluation of Potential Biomarkers for Targeted Intraoperative Fluorescence Imaging in Endometriosis: Towards Optimizing Surgical Treatment

**DOI:** 10.1007/s43032-024-01715-4

**Published:** 2024-10-07

**Authors:** Fokkedien H. M. P. Tummers, Rozemarijn de Koning, Maria K. Bazelmans, Frank Willem Jansen, Mathijs D. Blikkendaal, Ronald L. P. van Vlierberghe, Alexander L. Vahrmeijer, Hans Marten Hazelbag, Peter J. K. Kuppen

**Affiliations:** 1 Department of Gynecology, Leiden University Medical Center, Leiden, The Netherlands; 2 Department of Surgery, Leiden University Medical Center, Leiden, The Netherlands; 3 Department of Biomechanical Engineering, Delft University of Technology, Delft, The Netherlands; 4 Endometriose in Balans, Haaglanden Medical Center, The Hague, The Netherlands; 5 Nederlandse Endometriose Kliniek, Reinier de Graaf Hospital, Delft, The Netherlands; 6 Department of Pathology, Haaglanden Medical Center, The Hague, The Netherlands

**Keywords:** Endometriosis, Fluorescence-guided surgery, Immunohistochemistry, Biomarkers

## Abstract

Surgical intervention for endometriosis is an important treatment modality, yet incomplete resection resulting from poor visibility of affected tissue and consequently recurrence of disease remains a prevalent challenge. Intra-operative visualization of endometriosis, enabling fluorescence-guided surgery (FGS), could help to optimize surgical treatment. A biomarker, upregulated in endometriosis compared to adjacent tissue, is required to use as a target for FGS. Immunohistochemistry was used to evaluate protein expression of a selection of previously identified potential biomarkers. Ten biomarkers were stained in a large cohort of 84 tissues, both deep and peritoneal endometriosis and tissue without endometriosis, all from patients with confirmed endometriosis. MMP11 and VCAN showed the largest upregulation in endometriosis compared to adjacent tissue and showed a membranous or extracellular staining pattern. MMP11 is a promising target for glandular and stromal visualization, VCAN for stromal visualization only. For both biomarkers, upregulation was high in both peritoneal and deep endometriosis and for patients with and without hormonal medication. Other stained biomarkers showed non-beneficial characteristics based on staining pattern or upregulation. Analysis of all endometriosis samples showed that combined glandular and stromal targeting is expected to result in optimal visualization of endometriosis. Further research is needed to determine whether targeting one biomarker is sufficient for this goal, or if dual targeting is necessary. Development of clinical tracers for VCAN and MMP11 is necessary.

**Supplementary Information:**

The online version contains supplementary material available at 10.1007/s43032-024-01715-4.

## Introduction

Endometriosis, a disease affecting 10% of all reproductive-aged women, is defined as a hormone-dependent disease [1], characterized by the presence of endometrial-like tissue, including glands and stromal tissue, outside of the uterine cavity [2]. The endometrial like tissue is frequently accompanied by fibrosis [3]. Endometriosis is classified in three phenotypes: peritoneal endometriosis (PE), ovarian endometrioma (OMA) and deep endometriosis (DE). The foremost clinical manifestations encompass dysmenorrhea, pelvic pain, infertility, physical and emotional problems, contributing to significant morbidity and social impact [4]. The management of endometriosis remains a complex task, relying mostly on hormonal therapy and surgery [5, 6]. Nevertheless, up to 35% of patients needs repeated surgery, due to recurrence of symptoms or disease [7]. Incomplete removal of endometriosis stands out as a potential risk factor for recurrence [7, 8]. Accurate intra-operative identification of PE and DE poses a considerable clinical challenge, in contrast to clearly detectable OMA.

Intra-operative fluorescence-guided surgery (FGS) is an imaging technique, that utilizes fluorescent tracers for intra-operative tissue identification by creating a contrast between the tissue of interest and the surrounding tissue. As near-infrared (NIR) light is used, combined with a NIR camera, and visualization on a screen, the visibility of the surgical field is not altered. To use a targeted fluorescent tracer, a biomarker is needed to be used as a specific target, which should be upregulated in endometriosis compared to surrounding tissue. Additionally, the expressed location of the target should preferentially be membranous or extracellular, to enable the tracer to optimally reach the target [9]. The targeted tracer, consisting of an antibody or other structure conjugated to a NIR-fluorophore, can be administered intravenously or topically, depending on the location of the target tissue [9, 10].

Identifying these biomarkers is an important step in the road towards targeted endometriosis imaging. Our research group previously published a new approach to select potential targets, based on transcriptomics analysis [11]. This resulted in 29 potential targets ranked by a Target Selection Criteria (TASC) score. The current study evaluates the potential of these targets by studying protein expression using immunohistochemistry (IHC) in endometriosis and relevant surrounding tissue, i.e. peritoneum, bowel and bladder. Due to feasibility, a diverse sample of 10 of the 29 potential targets was chosen to validate. The goal was to stain the five most potential targets, and five targets with a lower ranking to use as a protein expression evaluation of the abovementioned approach. The most potential target, FOLR1 (rank 1) was chosen not to analyze, as protein expression was already validated in endometriosis and relevant surrounding tissue [12]. Therefore, the next five targets (rank 2–6) were all immunohistochemically analyzed, being CXCL8, MMP3, MMP7, IL1B, MMP10. Additionally, IGFBP1 (rank 8) CDH2 (rank 10), MMP11 (rank 12), PAEP (rank 16), VCAN (rank 17) were analyzed. These last five targets were chosen based on a combination of ranking, already available IHC studies, availability of antibodies and diverse characteristics of targets.

This study aims to validate targets on protein level to use for intra-operative FGS in endometriosis surgery by using immunohistochemistry.

## Materials and Methods

### Patient and Tissue Selection

Medical records and tissue specimens were retrospectively reviewed from patients who underwent endometriosis surgery in Endometriose in Balans, Haaglanden Medical Center between January 2019 and February 2021. Formalin-fixed paraffin-embedded (FFPE) endometriosis and non-endometriosis tissue specimens were included for analysis. Endometriosis specimens were identified as specimens with histopathological confirmed endometriosis by a pathologist, specialized in gynecology. Histologically confirmed non-endometriosis specimens from the same patients were identified as well. These could be whole specimens (i.e. uterus), or part of a specimen (final endometriosis-free parts of bowel segmental resection). For further analysis, these specimens are identified as ‘healthy specimens’. Based on the surgical and pathological reports, endometriosis specimens were categorized as PE or DE and the anatomical location was reported. Based on similar experiments, a desired minimum of around 20 samples per group was chosen, with the possibility of using multiple specimens per patients. Therefore, 60 patients were asked for informed consent, taking non-responders into account. Additionally, patient and tissue characteristics were collected from medical records, including age, parity, medication use. The revised American Society for Reproductive Medicine Classification (rASRM) stage and ENZIAN score (ENZIAN classification system), both classification systems for endometriosis, are included in the characteristics [13].

### Ethics Approval and Informed Consent

The study protocol was approved by the Medical Ethical Committee Leiden Delft The Hague (METC LDD, B21.033). Tissue specimens were collected from patients who provided written informed consent.

### Antibodies

Expression of ten biomarkers was immunohistochemically analyzed, being VCAN, IGFBP1, MMP10, MMP11, CDH2, PAEP, IL1B, CXCL8, MMP3, MMP7. The antibodies used for the immunohistochemical stainings are shown in Supplementary Table 1.

### Immunohistochemistry

FFPE tissue blocks were collected from the Pathology Department of Haaglanden Medical Center. Tissue sections of 4 µm were obtained. Sections of all tissue specimens were stained with hematoxylin and eosin (H&E) as well as with individual antibodies. For preparation of IHC staining, the slides were deparaffinized with xylene and rehydrated in serially diluted ethanol solutions (100%-70%-50%), followed by demineralized water and Phosphate Buffer Solution (PBS) according to standard protocols. For H&E, the slides were incubated for 5 minutes in hematoxylin, incubated with eosin for one minute, and covered with Pertex, according to a standard protocol. For immunohistochemical staining of the tissue sections with the antibodies, endogenous peroxidase activity was blocked by incubation in 0.3% hydrogen peroxidase in PBS for 20 min. Antigen retrieval was performed by heat induction at 95°C using PT Link (Dako, Agilent Technologies, Santa Clara, United States) with low-pH Envision FLEX target retrieval solution (pH 6.0, citrate buffer, Dako) or high-pH Envision FLEX target retrieval solution (pH 9.0, citrate buffer, Dako) (Supplemental Table 1).

Following antigen retrieval, the tissue sections were incubated overnight with the primary antibodies in 100 µL at room temperature. To achieve the most comparable staining results throughout the cohort, the use of one vial per antibody was desired. Therefore, for some antibodies less than 100 µL was used (25 of 50 µL), which is noted in Supplemental Table 1. For these antibodies, pre-testing showed no relevant difference between 100 µL without cover slip and respectively 25 or 50 µL with cover slip for those antibodies.

The slides were washed with PBS, followed by incubation with secondary antibodies for 30 minutes at room temperature (anti-mouse or anti-rabbit Envision/HRP, see Supplementary Table 1). After additional washing, the staining was visualized with 3,3-diaminobenizidine tetrahydrochloride solution (DAKO, Glustrup, Denmark) at room temperature for 10 minutes and counterstained with hematoxylin for 30 seconds. Finally, the tissue sections were dehydrated and mounted in Pertex (Histolab Products, Vasta Frolunda, Sweden).

An optimal dilution was pre-determined for all antibodies, using positive controls and endometriosis tissue specimens (Supplementary Table 1) and subsequently used for the entire cohort. All stained sections were scanned using the 3D Histech scanner (3D-Histech, Panoramic scanner 250).

### Analysis

In the H&E slides, endometriosis areas were annotated. Epithelial glands and stroma were annotated separately. In case of doubt, a pathologist, specialized in gynaecology (HMH) was consulted. Evaluation of immunohistochemical staining was performed by using a scoring system. This system comprised the proportion score (PS) and the intensity score (IS) which were multiplied by each other and resulted in the total immunostaining score (TIS) [14]. PS indicates the positively stained percentage of the tissue of interest and ranges between 0 and 4 (0=none, 1:>0% and <10%, 2:10-50%, 3:51-80%, 4:>80%). IS indicates the overall intensity of staining (0=none, 1=weak, 2=moderate, 3=strong). Multiplying IS and PS together results in TIS, with a maximum value of 12 (0=no expression, 1-4=weak expression, 6-8=mediate expression, 9-12=intense expression). The scoring was performed blindly and independently by two observers (FHMPT and RdK), after which a final agreement score was determined together for all slides. These scores were calculated for three tissue types in endometriosis specimens; the endometrial gland ducts, the stromal cells and adjacent tissue. In specimens without endometriosis (healthy specimens), the whole tissue was scored as healthy tissue. If adjacent or healthy tissue showed positive staining, the type of tissue showing expression was noted, additional to the TIS. In adjacent or healthy tissue, expression of biomarkers in bowel and tubal mucosa were not included in TIS. These tissue types were considered less relevant as intra-operatively the serosal side of bowel and tubes are visualized and not the mucosal side. However, positive staining was noted, so it could be included in the evaluation of the biomarker. Results for pattern of expression, differences between endometriosis subtypes and location of expression in the cells affected were analyzed for all biomarkers as well.

### Statistical Analysis

Statistical analysis was performed using SPSS version 29.0 software for Windows (SPSS, IBM Corporation, Somer NY, USA). Linear mixed model was used to analyze the difference in TIS (ΔTIS) between glands, stroma and adjacent tissue within specimens, and for adjacent and healthy tissue between specimens, including a subject factor as some patients provided multiple specimens. Subanalyses with linear mixed models were performed to differentiate the scoring differences between endometriosis subtype and hormonal medication use. Weighted kappa was calculated for inter-observer agreement between the two independent observers. Results were considered statistically significant at the level of P<0.05. GraphPad Prism 8 (GraphPad, Software, Inc, La Jolla CA, USA) was used to acquire the scatterplots.

## Results

### Patient and Tissue Characteristics

40 patients provided informed consent, providing 84 specimens, with 22 tissues of PE, 44 of DE and 18 healthy tissues. Table 1 shows a summary of patient characteristics and Table 2 a summary of specimen characteristics. The mean age of patients was 35.8 years and 72.5 % were nulliparous. 47.5% of the patients did not use hormonal mediation, and they showed a various cohort based rASRM and ENZIAN stages. The tissue samples were collected from various locations (Table 2), including bladder, pelvic wall, rectum, uterosacral ligaments and vaginal tissue. Patients provided between one and seven tissue samples.

**Table 1:** Summary of patient characteristics

Characteristics	Subgroup	Mean(SD) or n(%)
Age		35.8 (7.2)
Parity	*0*	29 (72.5)
	*1*	3 (7.5)
	*2*	7 (17.5)
	*3*	1 (2.5)
Previous hysterectomy	*Yes*	1 (2.5)
	*No*	39 (97.5)
Postmenopausal	*Yes*	0 (0)
	*No*	40 (100)
Hormonal medication*	*Combined oral contraceptive*	10 (25)
	*Progesterone oral*	5 (12.5)
	*Progesterone intra-uterine device*	6 (15)
	*Gonadotropin-releasing hormone agonist*	3 (7.5)
	*Aromatase inhibitor*	0 (0)
	*Other*	0 (0)
	*No medication*	19 (47.5)
rASRM stage**	*Stage 1*	9 (22.5)
	*Stage 2*	8 (20)
	*Stage 3*	12 (30)
	*Stage 4*	10 (25)
	*Not registered*	1 (2.5)
ENZIAN***	*A1*	1 (2.5)
	*A2*	3 (7.5)
	*A3*	4 (10)
	*B1*	1 (2.5)
	*B2*	15 (37.5)
	*B3*	8 (20)
	*C1*	3 (7.5)
	*C2*	6 (15)
	*C3*	4 (10)
	*FA*	19 (47.5)
	*FB*	7 (17.5)
	*FU*	11 (27.5)
	*FI*	8 (20)
	*FO*	3 (7.5)

^*^Patients can use more than 1 type of medication, therefore the total adds up to more than 40. ** rASRM: revised American Society for Reproductive Medicine classification system. ***ENZIAN: ENZIAN classification system

**Table 2:** Summary of tissue sample characteristics

Characteristics	*Subgroup*	N (%)			
Endometriosis type	*Peritoneal endometriosis*	22 (26.2)			
	*Deep endometriosis*	44 (52.4)			
	*Healthy tissue*	18 (21.4)			
Hormonal medication	*Hormonal medication*	44 (52.4)			
	*No hormonal medication*	40 (47.6)			
Anatomical locations		*Peritoneal endometriosis*	*Deep endometriosis*	*Healthy tissue*	*Total (%)*
	*Appendix*	0	2	1	3 (3.6)
	*Pelvic wall*	8	4	0	12 (14.3)
	*Bladder*	4	6	1	11 (13.1)
	*Diaphragm*	0	1	0	1 (1.2)
	*Para urethral*	0	5	0	5 (6.0)
	*Rectum*	1	8	3	12 (14.3)
	*Uterosacral ligament*	3	5	0	8 (9.5)
	*Sigmoid colon*	0	4	3	7 (8.3)
	*Ureter*	1	2	0	3 (3.6)
	*Vagina*	0	6	0	6 (7.1)
	*Abdominal wall*	1	0	0	1 (1.2)
	*Douglas*	1	0	0	1 (1.2)
	*Broad ligament*	1	0	0	1 (1.2)
	*Para rectal*	1	0	0	1 (1.2)
	*Round ligament*	1	0	1	2 (2.4)
	*Adhesion*	0	0	1	1 (1.2)
	*Peritoneum*	0	0	2	2 (2.4)
	*Fallopian tube*	0	1	3	4 (4.8)
	*Uterus*	0	0	3	3 (3.6)

Anatomical location describes the location of the tissue sample, as described in the surgical report

### Biomarkers

**Fig. 1:**
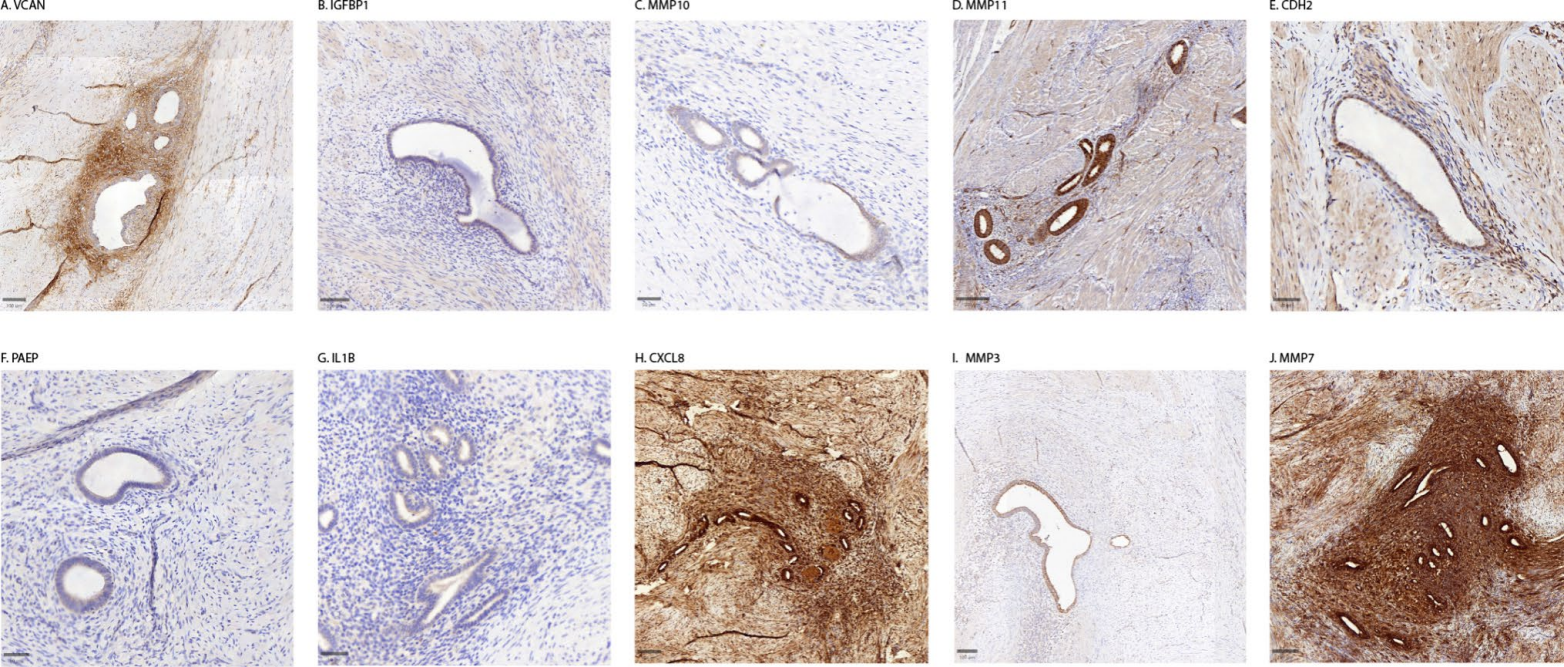
Representative immunohistochemical stainings of all biomarkers in endometriosis tissues. Location of tissue samples: Sigmoid (**A**,**D**,**I**), Vagina (**B**,**C**,**F**,**G**,**H**,**J**), Bladder (**E**). All stainings show deep endometriosis

Figure 1 shows representative staining of all biomarkers. Firstly, no statistically significant difference was observed between staining of adjacent tissue and healthy specimens for all biomarkers (Supplementary Table 2). Therefore, all TIS differences were calculated between glands and stroma versus adjacent tissue. The inter-observer agreement was substantial (weighted kappa = 0.629, *p*<0,001). For the TIS scores that did not match, a final agreement score was decided on by the two observers. Table 3 shows an overview of staining characteristics per biomarker and supplementary Table 2 shows the detailed results of all analyses.

**Table 3:** Overview of characteristics of all biomarkers

Target	Staining pattern glands	Staining pattern stroma	ΔTIS^∞^ glands versus adjacent tissue	ΔTIS^∞^ stroma versus adjacent tissue	Main advantage of biomarker	Main disadvantage of biomarker
VCAN	Cytoplasmic	Extracellular	2.06*	2.56*	Extracellular expression in stroma	27% of tissues showed no or weak expression in stroma.
IGFBP1	Cytoplasmic	No staining	2.74*	-2.87*	-	Cytoplasmic expression in glands. 41% of tissues showed weak expression in glands.
MMP10	Cytoplasmic	No staining	-0.24	-1.13*	-	Low expression in glands Cytoplasmic expression glands.
MMP11	Membranous, cytoplasmic	Extracellular	4.13*	2.54*	Large ΔTIS for glands Both glands and stroma show upregulation. Membranous staining glands, extracellular staining stroma	-
CDH2	Cytoplasmic, nucleus	Extracellular	3.99*	1.33	Large ΔTIS for glands Extracellular expression in stroma	Cytoplasmic expression in glands
PAEP	Cytoplasmic	No staining	0.33	-0.87*	-	Weak expression in endometriosis Cytoplasmic expression in glands
IL1B	Cytoplasmic	No staining	1.43*	-0.32*	-	Cytoplasmic expression in glands. 57% of tissues showed weak expression
CXCL8	Cytoplasmic, nucleus	Extracellular, nucleus	1.82*	0.99*	Extracellular expression in stroma	High expression in surrounding tissue. Cytoplasmic expression in glands.
MMP3	Cytoplasmic	No staining	6.38*	-2.55*	Large ΔTIS for glands 70% of tissues showed intense expression	Cytoplasmic expression in glands.
MMP7	Cytoplasmic	Extracellular	2.04*	0.17	-	High expression in surrounding tissue. Cytoplasmic expression in glands.

^**∞**^ΔTIS: difference in Total Immunostaining Score, observed in immunohistochemical staining. *statistically significant, *p*<0.05

#### Versican (VCAN)

**Fig. 2:**
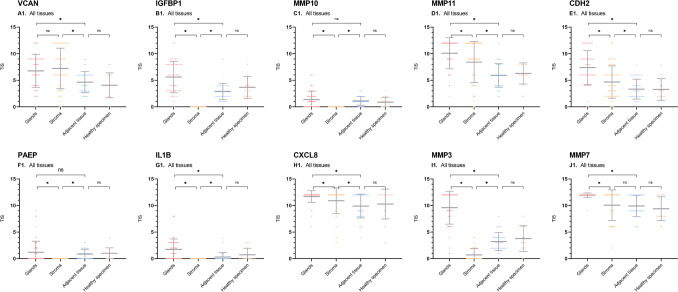
Scatterplots of total immunostaining score (TIS) for all biomarkers in endometriosis. TIS was analysed after immunohistochemical staining. Scatterplots show analysis of all tissues. *= statistical significance *p*<0.05. ns= non significance. Dots represent individual samples. Bars show means with confidence intervals

**Fig. 3:**
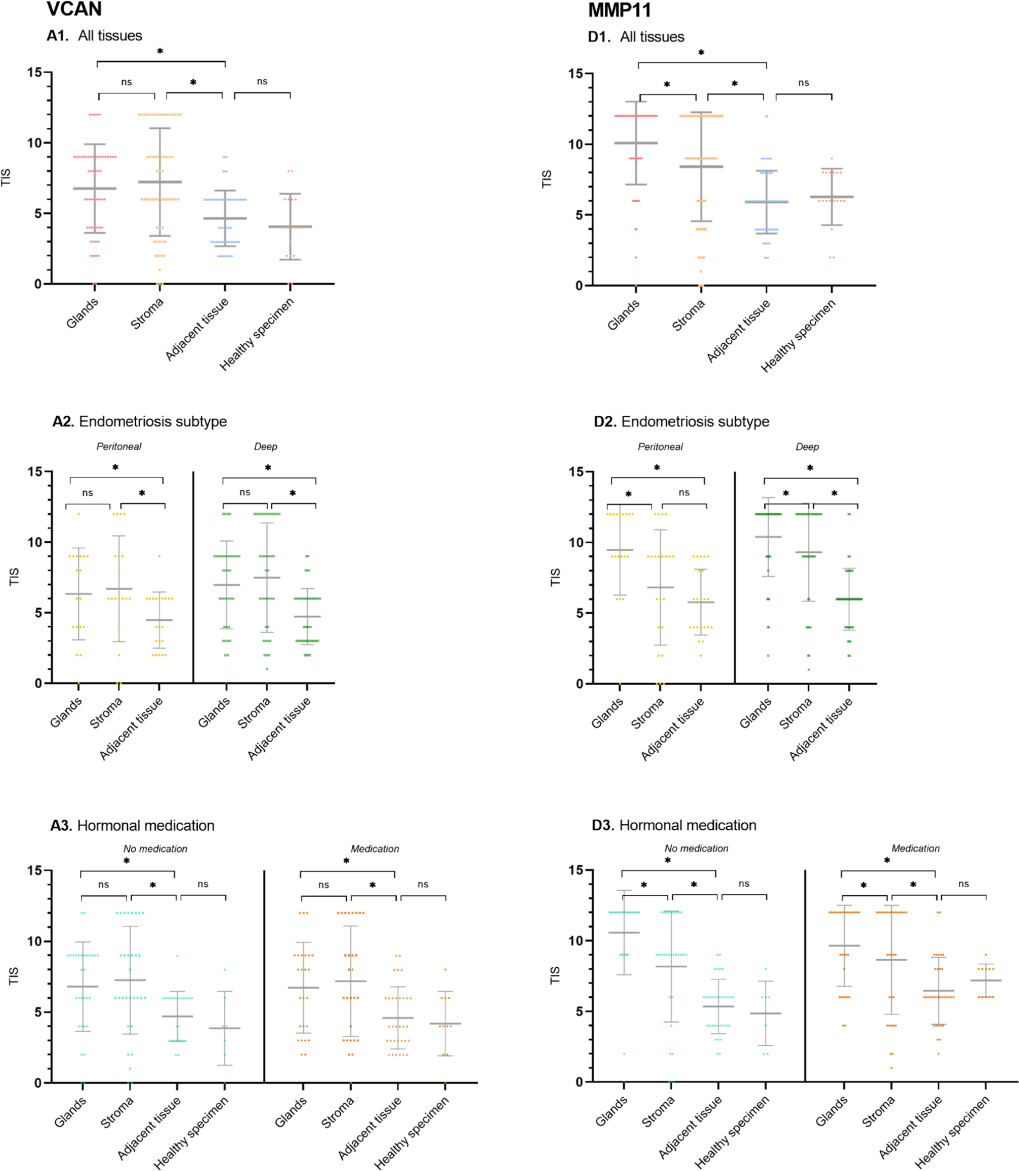
Scatterplots of total immunostaining score (TIS) for subanalyses for VCAN (A1-3) and MMP11 (D1-3) in endometriosis. TIS was analysed after immunohistochemical staining. Scatterplots show sub analyses of endometriosis subtype (2) and hormonal medication use (3). *= statistical significance *p*<0.05. ns= non significance. Dots represent individual samples. Bars show means with confidence intervals

**Fig. 4:**
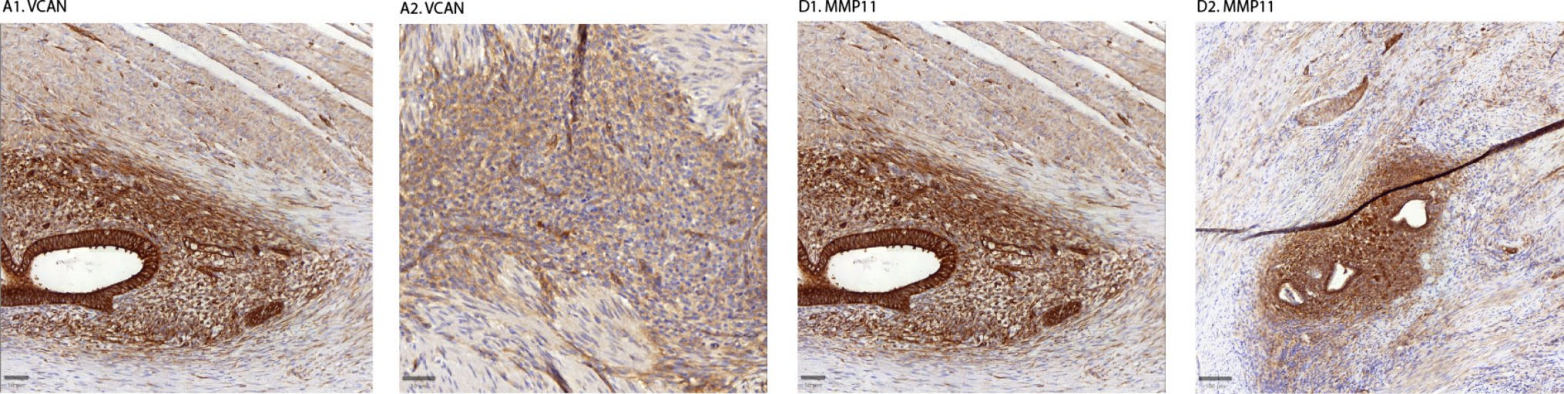
Staining pattern VCAN (A1-2) and MMP11 (D1-2). Staining pictures zoomed to focus on staining pattern. A1 and D1 is focused on glandular staining pattern, A2 and D2 are focused on stromal staining pattern. Location of tissue samples: Sigmoid colon (A1-2, D1), Vagina (D2)

Our analysis showed upregulation of VCAN in glands compared to adjacent tissue (ΔTIS 2.06, *p*<0.001), and in stroma compared to surrounding tissue (ΔTIS 2.56, *p*<0.001) (Figs. 1A and 2A1, Table 4). 41% of the slides showed intense staining of glands and 44% intense staining of stroma. This upregulation showed consistency in subgroup analysis for endometriosis type and hormonal medication use (Fig. 3A1-3). The staining showed a cytoplasmic staining pattern of glands and extracellular staining pattern in stroma (Fig. 4A1-2). Additionally, in adjacent and healthy tissue, smooth muscle cells, loose connective tissue and vessel walls showed weak or mediate staining.

**Table 4:** Statistical analysis of total immunostaining score (TIS) for VCAN

Target	Subanalysis		ΔTIS^∞^ Glands vs adjacent tissue	P value	ΔTIS^∞^ Stroma vs adjacent tissue	P value	ΔTIS^∞^ Glands vs stroma	*P* value	ΔTIS^∞^ Adjacent tissue vs healthy specimen	P value
VCAN	All endometriosis types		2.06	<0.001	2.56	<0.001	-0.50	0.207	0.43	0.324
	Subtypes	Peritoneal	1.92	0.018	2.28	0.006	-0.36	0.599		
		Deep	2.13	<0.001	2.70	<0.001	-0.57	0.241		
	Hormonal medication	Hormonal medication	2.09	0.002	2.53	<0.001	-0.44	0.457	0.46	0.653
		No hormonal medication	2.03	0.002	2.58	<0.001	-0.55	0.310	1.17	0.329

#### Insulin-like Growth Factor-binding Protein 1 (IGFBP1)

Glands showed upregulation for IGFBP1 in contrast to adjacent tissue (ΔTIS 2.74, *p*<0.001) (Figs. 1B and 2B1), with almost 80% showing weak or moderate expression. The upregulation was more distinct in patients without hormonal medication than in patients with hormonal medication (ΔTIS 3.64, *p*<0.001 versus 1.84, *p*<0.001). Stroma showed no expression of IGFBP1 and therefore a lower expression was observed than in adjacent tissue (ΔTIS -2.87, *p*<0.001). Endometrial glands showed a cytoplasmic staining pattern. In adjacent and healthy tissue, vessel walls and loose connective tissue showed weak staining.

#### Matrix Metalloprotein 10 (MMP10)

Analysis showed only weak expression of MMP10 in endometriosis (Fig. 2C1), with both glands and stroma showing lower expression than adjacent tissue (ΔTIS -0.24, *p*=0.197 and -1.13, *p*<0.001, Fig. 1C). Staining pattern was cytoplasmic for glands, stroma showed no staining. Adjacent and healthy tissue showed weak expression, mostly for vessel walls and loose connective tissue.

#### Matrix Metalloprotein 11 (MMP11)

For MMP11, glands showed upregulation compared to adjacent tissue (ΔTIS 4.13, *p*<0.001), with 81% of all slides showing intense expression (Figs. 1D and 2D1, Table 5). Additionally, stroma was upregulated in contrast to adjacent tissue (ΔTIS 2.54, *p*<0.001). The upregulation was larger for patients without hormonal medication compared to patients with hormonal medication (ΔTIS 4.97, *p*<0.001 versus 3.36, *p*<0.001 for glands) and larger in DE in contrast to peritoneal endometriosis (ΔTIS 4.54, *p*<0.001 versus 3.38, *p*<0.001 for glands and 3.38, *p*<0.001 versus 1.05, *p*=0.190 for stroma, Fig. 3D1-3). Glands showed both a membranous and cytoplasmic staining pattern, stroma an extracellular pattern (Fig. 4D1-2). Adjacent and healthy tissue, especially vessel walls, tubal epithelium and bowel epithelium showed mostly weak and mediate expression.

**Table 5:** Statistical analysis of total immunostaining score (TIS) for MMP11

Target	Subanalysis		ΔTIS^∞^ Glands vs adjacent tissue	P value	ΔTIS^∞^ Stroma vs adjacent tissue	P value	ΔTIS^∞^ Glands vs stroma	*P* value	ΔTIS^∞^ Adjacent tissue vs healthy specimen	P value
MMP11	All endometriosis types		4.13	<0.001	2.54	<0.001	1.59	<0.001	-0.20	0.792
	Subtypes	Peritoneal	3.38	<0.001	1.05	0.190	2.34	0.001		
		Deep	4.54	<0.001	3.38	<0.001	1.16	0.013		
	Hormonal medication	Hormonal medication	3.36	<0.001	2.22	0.001	1.15	0.032	-0.89	0.369
		No hormonal medication	4.97	<0.001	2.84	<0.001	2.12	<0.001	1.02	0.382

#### Cadherin-2 (CDH2)

Glands were upregulated for CDH2 compared to adjacent tissue (ΔTIS 3.99, *p*<0.001, Figs. 1E and 2E1), with intense expression in 43.1% of the tissues. Stroma was slightly upregulated versus adjacent tissue (ΔTIS 1.33, *p*<0.001). Glands showed a cytoplasmic and nucleic staining pattern, stroma an extracellular pattern. Adjacent tissue showed weak staining in smooth muscle cells and vessel walls. Bowel and tubal epithelium showed positive staining.

#### Progestagen Associated Endometrial Protein (PAEP)

PAEP staining was mostly absent or weak with only small differences between endometriotic glands and adjacent tissue (ΔTIS 0.33, *p*=0.147, Figs. 1F and 2F1). Stroma showed no staining. Limited sub analyses showed statistically significant, but only small or negative (higher expression in adjacent tissue compared to glands or stroma) TIS differences, as is shown in Supplemental Table 2F. PAEP showed a cytoplasmic staining pattern in glands.

#### Interleukin-1β (IL-1β)

IL1B staining showed slight overexpression of glands versus adjacent tissue (ΔTIS 1.43, *p*<0.001), with weak expression in 57% of the tissues (Fig. 1G). Stroma showed no staining in all tissues (Fig. 2G1). Glands showed a cytoplasmic staining pattern. There was no expression of IL1B on relevant adjacent and healthy tissue.

### CXC Motif Chemokine Ligand 8 (CXCL8)

CXCL8 was overexpressed in glands compared to adjacent tissue (ΔTIS 1.82, *p*<0.001) and in stroma compared to adjacent tissue (ΔTIS 0.99, p=0.005, Fig. 1H). Almost all tissues showed strong expression for both glands and stroma (Fig. 2H1). However, adjacent and healthy tissue also showed moderate and strong expression, resulting in a small TIS difference. Glands showed a cytoplasmic staining pattern, stroma an extracellular and nucleic pattern.

#### Matrix Metalloprotein 3 (MMP3)

Glands were overexpressed for MMP3 in contrast to surrounding tissue (ΔTIS 6.38, *p*<0.001), consistent for all sub analyses, with 70% of all tissues showing intense expression in glands (Figs. 1I and 2I1). The glands showed a cytoplasmic staining pattern. Stroma showed no staining. In adjacent and healthy tissue, vessel walls were stained.

#### Matrix Metalloprotein 7 (MMP7)

MMP7 was overexpressed in glands compared to surrounding tissue (ΔTIS 2.04, *p*<0.001, Figs. 1J and 2J1). It showed a cytoplasmic staining pattern in glands and an extracellular pattern in stroma. All tissue samples showed intense expression of MMP7 in glands and 76% of the tissues showed intense expression in stroma. Adjacent and healthy tissues showed intense expression as well, especially vessel walls, bowel and tubal epithelium.

## Discussion

### Main Findings

The present study showed the next step towards intra-operative fluorescence-guided surgery for endometriosis. Potential biomarkers to use as a target for fluorescent imaging, previously selected using transcriptomic analysis, were validated to assess protein expression in endometriosis and relevant adjacent tissue using immunohistochemistry. MMP11 showed the largest overexpression in glands compared to adjacent tissue, and additionally showed stromal expression. VCAN showed the largest overexpression in stroma. Both biomarkers showed a beneficial staining pattern, meaning membranous or extracellular.

### Potential Biomarkers

In our analysis, MMP11 showed beneficial characteristics for intra-operative visualization. It showed a large upregulation in endometrial glands compared to adjacent tissues, combined with a membranous staining pattern. Stroma showed large upregulation with an extracellular staining pattern.

MMP11 encodes for an enzyme, that cleaves alpha 1-proteinase inhibitor but weakly degrades structural proteins of the extracellular matrix [15]. MMPs are involved in the breakdown of extracellular matrix in normal physiological processes, such as embryonic development, reproduction, and tissue remodeling [16]. MMPs, and MMP11 specifically, were expressed in several types of endometriosis and showed a higher expression level in ectopic endometrium compared to eutopic endometrium. [17]. Next to that, MMPs are also found to be involved in the decidualization of endometrial stroma [18–20]. MMP11 scored on the TASC score in our previous study due to literature showing diffuse upregulation of MMP11 in patients and a beneficial subcellular location [11].

Interestingly, MMP11 was anticipated to be extracellularly but exhibited distinct membranous staining in glands, additional to an extracellular pattern in stroma. The membranous location was supported by information from datasheets from other MMP11 antibodies and IHC analyses [21, 22]. Despite MMP11 not being classified as a membrane-type MMP, staining might suggest that post-translational modifications, such as glycosylation or phosphorylation, have influenced protein properties [23]. (Pre-)clinical studies are needed to show were MMP11 is visualized using a fluorescent tracer.

Focusing on medication use, upregulation was slightly stronger for patients with hormonal medication. The influence of hormones on MMPs is supported by literature showing that progesterone inhibition of endometrial MMPs during the secretory phase might be deficient in women with endometriosis [20] and that MMPs are influenced by changes in steroid hormone concentration levels [24].

Concentrating on endometriosis type, upregulation was slightly larger for DE. The larger upregulation of MMP11 in DE is supported by MMPs important role in fibrosis [25]. Fibrosis is thought to play an important role in PE, as well as an even more distinct role in DE [3], being the most fibrotic and infiltrated type of endometriosis [26]. This supports the presence of MMP11 in both subtypes of endometriosis, with a slightly higher upregulation of MMP11 in DE. Although small differences were observed in these sub analyses, upregulation of endometriosis was large in all subgroups. This indicates strong potential for MMP11 to use as a universal target for visualization of endometrial glands and stroma for endometriosis surgery.

In addition to MMP11, VCAN showed promising characteristics. It showed large upregulation in stroma compared to adjacent tissue, with an extracellular staining pattern. No relevant difference was seen within hormonal medication use or types of endometriosis, making VCAN a universal biomarker to use as a target for visualization of endometrial stroma.

VCAN might play a role in intercellular signaling and connecting cells with extracellular matrix [27]. It participates in cell adhesion and angiogenesis [28], it induces inflammation and is found to be upregulated in peritoneum of women with endometriosis compared to women without endometriosis [29]. Further, little is known regarding the correlation between VCAN and endometriosis. VCAN resulted in a high TASC score due to gene upregulation in both peritoneal and deep endometriosis, its association with fibrosis and low RNA expression in surrounding tissue [11].

For both MMP11 and VCAN, staining was observed in adjacent tissue (MMP11: vessel walls, VCAN: smooth muscle cells, loose connective tissue, vessel walls), however significant less than in endometriosis, resulting in high TIS differences. Human Protein Atlas shows on single cell level that vascular smooth muscle cells and endothelial cells show RNA expression for VCAN [30], and could therefore indicate specific staining. For MMP11 no RNA expression was observed [31] and no information was available for non-vascular smooth muscle cells and loose connective tissue. Protein expression is observed for VCAN in smooth muscle cells, connective tissue of bowel, bladder and vascular wall [32]. MMPs have shown a role in vascular remodeling [33], are secreted by smooth muscle cells [34], and might also be found in connective tissue [35]. This might suggest that staining in adjacent tissue could be specific. Further research with fluorescent tracers should focus on specificity of staining in adjacent tissue.

Although our previous analysis only selected biomarkers with a low RNA expression level (expressed in Transcripts per Million (TPM)) for fallopian tubes and bowel [11], both tubal and bowel mucosal epithelium stained positive for VCAN and MMP11. The used TPM values were based on tissue as a whole, being an average of all single cell types. For both MMP11 and VCAN, RNA expression on single cell level showed low TPM values [30, 31]. Therefore, staining might be non-specific. However, with clinical fluorescence use, mucosal staining is anticipated not to pose a significant issue since the bowel and tube will intra-operatively be observed from a serosal side of the organs.

### Non-potential Biomarkers

All other analyzed biomarkers showed less favorable combinations of characteristics. Although IGFBP1, CHD2, MMP3 and MMP7 exhibit a high expression compared to adjacent tissue, staining showed a cytoplasmic staining pattern. This suggests less favorable characteristics for the use as a target, as this would result in the need for internalization in the cell. Additionally, cytoplasmic location for expected extracellular located biomarkers could be due to cellular processing and secretion as it may be synthesized in the cytoplasm and then transported to the extracellular space. When the protein is actively secreted or undergoes cellular processing before being released, the cytoplasmic form might be more abundant and detectable by the antibody. Therefore some biomarkers might still have an extracellular location which was not observed in this analysis. Considering this, these biomarkers are not considered the most potential, but should not be completely excluded from future research.

MMP10, PAEP and IL1B revealed low expression in endometriosis, while showing a positive staining in positive control tissue, and are therefore not considered potential for further research. Although CXCL8 showed high expression in endometriosis, all adjacent tissue showed intense staining as well. This may arise from non-specific staining, however given its interleukin nature, this biomarker may manifest ubiquitous high expression. Future research could also focus on the potential of CXCL8.

### Validation of Target Identification Method

Previous analysis identified 29 potential targets [11], of which 10 were analyzed in this study. In this previous analysis, a new promising method was used to broaden the identification of candidate targets, combining transcriptomic analysis of publicly available data, with target selection criteria. The current study showed that this method, indeed, resulted in potential targets for FGS. However, multiple selected potential targets, including highly ranked targets, showed less favorable characteristics after protein expression validation. This underscores that RNA expression does not invariably translate directly into protein expression [36]. Still, the used method is considered valuable, as it could result in new potential targets, which would not have been identified with conventional methods [11]. However, validation of potential targets for protein expression is essential.

### Clinical Perspectives

FGS is thought to be a promising surgical modality for endometriosis surgery. Most new laparoscopy and robotic devices have a fluorescence function. Targeted FGS in endometriosis could be used for several purposes. Mainly, it could be used to enhance identification of endometriosis spots. Additionally, it could be used to determine resection planes by improved identification, to obtain complete excision with enhanced precision and safety. In this study, we focused on visualization of endometriosis itself, however also taking association of the studied biomarker with fibrosis into account [11]. Fibrosis is present in both peritoneal and to more extent in deep endometriosis and develops in reaction to the presence of endometriosis due to recurrent injury and repair [37]. Especially in deep endometriosis, this fibrosis can also be a cause of pain symptoms. However, in contrast to endometriosis, the visualization of fibrosis is considered to be a less significant problem during surgery, as conventional white-light visualization and especially haptic feedback provides valuable information regarding the location. Therefore, by using FGS in combination with white-light visualization and haptic feedback, a more complete and precise removal of endometriosis and associated fibrosis could potentially be achieved.

Studying the tissues showed insightful characteristics of endometriosis, with a ‘patchy’ pattern throughout the tissue. Therefore it could be challenging to visualize endometriosis intra-operatively, as this may result in scattering of the light. Future studies need to focus on clinical behavior of endometriosis during FGS. Additionally, in some nodules, only glands or stromal tissue might be present, which makes concurrent targeting of these structures valuable. Furthermore, glands constitute a small component of the nodule, which points out the importance of stromal visualization. A fluorescent tracer targeting a biomarker staining both glands and stroma, or a dual tracer is thought to be the optimal way for intra-operative visualization of endometriosis.

### Future Research

The potential targets identified in this study, together with previously identified FOLR1 [11, 12], should be analyzed further preclinically by using fluorescent tracers. Pre-clinical models of endometriosis are scarce and challenging. Increasing the predictive value of preclinical models is a hurdle for endometriosis scientists [38]. Research is ongoing on new options to reconstruct human endometriosis to improve translational research [38]. Post-operative ex-vivo endometriosis tissue could be used to validate fluorescent tracers.

Unfortunately, no clinical tracers for both MMP11 and VCAN are available yet. However, numerous fluorescent-labelled or radio-labelled MMP inhibitors for other MMPs have been developed as imaging agents for clinical trials, but additional research is required [39]. However, this knowledge might accelerate the development of a clinical tracer for MMP11. For VCAN, research in this field is ongoing as targeting VCAN is considered a therapeutic potential for multiple diseases [40].

### Strengths

In this study, a large and various cohort of endometriosis samples and healthy specimens was collected. Additionally, the healthy specimens were collected from the same patients. This makes it optimally translatable to clinical use, as the intra-operative differentiation between healthy and endometriosis tissue needs to be made within the same patient. Secondly, multiple biomarkers were tested based on previous selection [11], which resulted in potential targets but concurrently validates the previous new method, using publicly available data, which offers future research potential for other diseases.

### Limitations

As mentioned previously, our analysis revealed occasionally unexpected observed staining patterns and some non-specific staining. As a result, some biomarkers exhibited non-beneficial characteristics based on these observations. However, these biomarkers might still have a potential to use as a target, and could be analyzed further if MMP11 and VCAN prove unsuitable in further research steps towards a fluorescent tracer.

## Conclusion

Immunohistochemical evaluation of ten potential biomarkers to use as a target in fluorescence guided surgery in endometriosis surgery showed MMP11 and VCAN to be the most promising biomarkers. MMP11 and VCAN showed the largest upregulation in endometriosis compared to adjacent tissue and showed a membranous or extracellular staining pattern. MMP11 is a promising target for glandular and stromal visualization, VCAN for stromal visualization only.

## Supplementary Information

Below is the link to the electronic supplementary material.

Supplementary file1 (DOCX 41 KB)
