# Supplementary material for: Immunohistochemical Evaluation of Potential Biomarkers for Targeted Intraoperative Fluorescence Imaging in Endometriosis: Towards Optimizing Surgical Treatment
Source: Reprod Sci. 2024 Oct 7;31(12):3705–18. doi: 10.1007/s43032-024-01715-4 (PMC11611954; doi:10.1007/s43032-024-01715-4)
Supplement: Supplementary file 1 — Supplementary file1 (DOCX 41 KB) [file 43032_2024_1715_MOESM1_ESM.docx]

| Target | Primary antibody | Concentration | Dilution | Monoclonal/Polyclonal | Antigen retrieval | Species | Positive control |
| --- | --- | --- | --- | --- | --- | --- | --- |
| VCAN | Recombinant Anti-VCAN, Ab177480  *Abcam* | 0.115 mg/ml | 1:400, 100 µl | Monoclonal | pH 9.0 | Rabbit | Bladder |
| IGFBP1 | IGFBP1, TA808733  *Thermofisher/OriGene* | 1 mg/ml | 1:400, 100 µl | Monoclonal | pH 9.0 | Mouse | Endometrium |
| MMP10 | Anti-MMP10, Ab56304  *Abcam* | 0.2 mg/ml | 1:150, 100 µl | Monoclonal | pH 6.0 | Mouse | Placenta |
| MMP11 | MMP11 Recombinant, MA5-32285  *Thermofisher/Invitrogen* | 1 mg/ml | 1:200, 100 µl | Monoclonal | pH 6.0 | Rabbit | Spleen |
| CHD2 | Recombinant anti-N Cadherin, Ab76011  *Abcam* | 0.097 mg/ml | 1:100, 100 µl | Monoclonal | pH 6.0 | Rabbit | Liver |
| PAEP | Anti-PAEP/Glycodelin, Ab17247  *Abcam* | 1 mg/ml | 1:50, 25 µl with coverslip | Monoclonal | pH 6.0 | Mouse | Endometrium |
| IL1B | Recombinant Monoclonal Antibody anti-IL-1 beta. JJ087-3  *Novusbio* | 1 mg/ml | 1:50, 50 µl with coverslip | Monoclonal | pH 9.0 | Rabbit | Kidney |
| CXCL8 | Anti-IL8 Antibody, PA5-114316  *ThermoFisher* | 1 mg/ml | 1:100, 100 µl | Polyclonal | pH 9.0 | Rabbit | Skeletal muscle |
| MMP3 | Recombinant anti-MMP3 antibody, Ab52915  *Abcam* | 0.386 mg/ml | 1:50, 25 µl with coverslip | Monoclonal | pH 9.0 | Rabbit | Liver |
| MMP7 | Anti-MMP7 antibody  Ab38996  *Abcam* | 1 mg/ml | 1:300, 100 µl | Polyclonal | pH 6.0 | Rabbit | Placenta |

Supplementary table 1. **Antibodies and immunohistochemical staining details**

| **Target** | **Subanalysis** |  | **ΔTIS^∞^ Glands vs adjacent tissue** | **P value** | **ΔTIS^∞^ Stroma vs adjacent tissue** | **P value** | **ΔTIS^∞^ Glands vs stroma** | **P value** | **ΔTIS^∞^ Adjacent tissue vs healthy specimen** | **P value** |
| --- | --- | --- | --- | --- | --- | --- | --- | --- | --- | --- |
| **VCAN** | All endometriosis types |  | 2.06 | <0.001 | 2.56 | <0.001 | -0.50 | 0.207 | 0.43 | 0.324 |
|  | Subtypes | Peritoneal | 1.92 | 0.018 | 2.28 | 0.006 | -0.36 | 0.599 |  |  |
|  |  | Deep | 2.13 | <0.001 | 2.70 | <0.001 | -0.57 | 0.241 |  |  |
|  | Hormonal medication | Hormonal medication | 2.09 | 0.002 | 2.53 | <0.001 | -0.44 | 0.457 | 0.46 | 0.653 |
|  |  | No hormonal medication | 2.03 | 0.002 | 2.58 | <0.001 | -0.55 | 0.310 | 1.17 | 0.329 |

Supplementary table 2A. **Statistical analysis of total immunostaining score for VCAN**

| **Target** | **Subanalysis** |  | **ΔTIS^∞^ Glands vs adjacent tissue** | **P value** | **ΔTIS^∞^ Stroma vs adjacent tissue** | **P value** | **ΔTIS^∞^ Glands vs stroma** | **P value** | **ΔTIS^∞^ Adjacent tissue vs healthy specimen** | **P value** |
| --- | --- | --- | --- | --- | --- | --- | --- | --- | --- | --- |
| **IGFBP1** | All endometriosis types |  | 2.74 | <0.001 | -2.87 | <0.001 | 5.61 | <0.001 | -0.76 | 0.124 |
|  | Subtypes | Peritoneal | 2.81 | <0.001 | -2.57 | <0.001 | 5.38 | <0.001 |  |  |
|  |  | Deep | 2.71 | <0.001 | -3.02 | <0.001 | 5.73 | <0.001 |  |  |
|  | Hormonal medication | Hormonal medication | 1.84 | <0.001 | -3.27 | <0.001 | 5.11 | <0.001 | -0.22 | 0.729 |
|  |  | No hormonal medication | 3.64 | <0.001 | -2.47 | <0.001 | 6.11 | <0.001 | -1.36 | 0.073 |

Supplementary table 2B **Statistical analysis of total immunostaining score for IGFBP1**

| **Target** | **Subanalysis** |  | **ΔTIS^∞^ Glands vs adjacent tissue** | **P value** | **ΔTIS^∞^ Stroma vs adjacent tissue** | **P value** | **ΔTIS^∞^ Glands vs stroma** | **P value** | **ΔTIS^∞^ Adjacent tissue vs healthy specimen** | **P value** |
| --- | --- | --- | --- | --- | --- | --- | --- | --- | --- | --- |
| **MMP10** | All endometriosis types |  | -0.24 | 0.197 | -1.13 | <0.001 | 1.37 | <0.001 | 0.24 | 0.372 |
|  | Subtypes | Peritoneal | 0.67 | 0.042 | -0.68 | <0.001 | 1.39 | <0.001 |  |  |
|  |  | Deep | 0.03 | 0.908 | -1.36 | <0.001 | 1.34 | <0.001 |  |  |
|  | Hormonal medication | Hormonal medication | -0.05 | 0.842 | -1.21 | <0.001 | 1.16 | <0.001 | 0.27 | 0.449 |
|  |  | No hormonal medication | 0.53 | 0.044 | -1.04 | <0.001 | 1.58 | <0.001 | 0.24 | 0.567 |

Supplementary table 2C. **Statistical analysis of total immunostaining score for MMP10**

| **Target** | **Subanalysis** |  | **ΔTIS^∞^ Glands vs adjacent tissue** | **P value** | **ΔTIS^∞^ Stroma vs adjacent tissue** | **P value** | **ΔTIS^∞^ Glands vs stroma** | **P value** | **ΔTIS^∞^ Adjacent tissue vs healthy specimen** | **P value** |
| --- | --- | --- | --- | --- | --- | --- | --- | --- | --- | --- |
| **MMP11** | All endometriosis types |  | 4.13 | <0.001 | 2.54 | <0.001 | 1.59 | <0.001 | -0.20 | 0.792 |
|  | Subtypes | Peritoneal | 3.38 | <0.001 | 1.05 | 0.190 | 2.34 | 0.001 |  |  |
|  |  | Deep | 4.54 | <0.001 | 3.38 | <0.001 | 1.16 | 0.013 |  |  |
|  | Hormonal medication | Hormonal medication | 3.36 | <0.001 | 2.22 | 0.001 | 1.15 | 0.032 | -0.89 | 0.369 |
|  |  | No hormonal medication | 4.97 | <0.001 | 2.84 | <0.001 | 2.12 | <0.001 | 1.02 | 0.382 |

Supplementary table 2D. **Statistical analysis of total immunostaining score for MMP11**

| **Target** | **Subanalysis** |  | **ΔTIS^∞^ Glands vs adjacent tissue** | **P value** | **ΔTIS^∞^ Stroma vs adjacent tissue** | **P value** | **ΔTIS^∞^ Glands vs stroma** | **P value** | **ΔTIS^∞^ Adjacent tissue vs healthy specimen** | **P value** |
| --- | --- | --- | --- | --- | --- | --- | --- | --- | --- | --- |
| **CDH2** | All endometriosis types |  | 3.99 | <0.001 | 1.33 | 0.003 | 2.67 | <0.001 | 0.14 | 0.845 |
|  | Subtypes | Peritoneal | 4.12 | <0.001 | 1.86 | 0.013 | 2.26 | <0.001 |  |  |
|  |  | Deep | 3.97 | <0.001 | 1.07 | 0.047 | 2.90 | <0.001 |  |  |
|  | Hormonal medication | Hormonal medication | 3.64 | <0.001 | 0.86 | 0.163 | 2.79 | <0.001 | 0.21 | 0.816 |
|  |  | No hormonal medication | 4.33 | <0.001 | 1.78 | <0.001 | 2.56 | <0.001 | 0.167 | 0.877 |

Supplementary table 2E. **Statistical analysis of total immunostaining score for CDH2**

| **Target** | **Subanalysis** |  | **ΔTIS^∞^ Glands vs adjacent tissue** | **P value** | **ΔTIS^∞^ Stroma vs adjacent tissue** | **P value** | **ΔTIS^∞^ Glands vs stroma** | **P value** | **ΔTIS^∞^ Adjacent tissue vs healthy specimen** | **P value** |
| --- | --- | --- | --- | --- | --- | --- | --- | --- | --- | --- |
| **PAEP** | All endometriosis types |  | 0.33 | 0.147 | -0.87 | <0.001 | 1.20 | <0.001 | -0.06 | 0.856 |
|  | Subtypes | Peritoneal | -0.045 | 0.905 | -0.27 | 0.128 | 0.23 | 0.572 |  |  |
|  |  | Deep | 0.56 | 0.045 | -1.18 | <0.001 | 1.75 | <0.001 |  |  |
|  | Hormonal medication | Hormonal medication | 0.95 | 0.002 | -1.04 | <0.001 | 1.99 | <0.001 | -0.30 | 0.475 |
|  |  | No hormonal medication | -0.29 | 0.350 | -0.69 | 0.024 | 0.40 | 0.221 | 0.28 | 0.570 |

Supplementary table 2F. **Statistical analysis of total immunostaining score for PAEP**

| **Target** | **Subanalysis** |  | **ΔTIS^∞^ Glands vs adjacent tissue** | **P value** | **ΔTIS^∞^ Stroma vs adjacent tissue** | **P value** | **ΔTIS^∞^ Glands vs stroma** | **P value** | **ΔTIS^∞^ Adjacent tissue vs healthy specimen** | **P value** |
| --- | --- | --- | --- | --- | --- | --- | --- | --- | --- | --- |
| **IL1B** | All endometriosis types |  | 1.43 | <0.001 | -0.32 | 0.006 | 1.75 | <0.001 | -0.37 | 0.249 |
|  | Subtypes | Peritoneal | 1.05 | 0.007 | -1.67*10^-16^ | 1.000 | 1.05 | 0.010 |  |  |
|  |  | Deep | 1.66 | <0.001 | -0.49 | <0.001 | 2.15 | <0.001 |  |  |
|  | Hormonal medication | Hormonal medication | 1.03 | 0.002 | -0.43 | 0.010 | 1.46 | <0.001 | -0.26 | 0.534 |
|  |  | No hormonal medication | 1.82 | <0.001 | -0.22 | 0.164 | 2.03 | <0.001 | -0.46 | 0.354 |

Supplementary table 2G. **Statistical analysis of total immunostaining score for IL1B**

| **Target** | **Subanalysis** |  | **ΔTIS^∞^ Glands vs adjacent tissue** | **P value** | **ΔTIS^∞^ Stroma vs adjacent tissue** | **P value** | **ΔTIS^∞^ Glands vs stroma** | **P value** | **ΔTIS^∞^ Adjacent tissue vs healthy specimen** | **P value** |
| --- | --- | --- | --- | --- | --- | --- | --- | --- | --- | --- |
| **CXCL8** | All endometriosis types |  | 1.82 | <0.001 | 0.99 | 0.005 | 0.83 | 0.004 | -0.14 | 0.797 |
|  | Subtypes | Peritoneal | 1.63 | 0.006 | 0.64 | 0.288 | 0.99 | 0.041 |  |  |
|  |  | Deep | 1.92 | <0.001 | 1.18 | 0.007 | 0.74 | 0.034 |  |  |
|  | Hormonal medication | Hormonal medication | 1.49 | 0.002 | 1.10 | 0.027 | 0.38 | 0.310 | -0.17 | 0.808 |
|  |  | No hormonal medication | 2.17 | <0.001 | 0.87 | 0.085 | 1.30 | 0.001 | 0.030 | 0.972 |

Supplementary table 2H. **Statistical analysis of total immunostaining score for CXCL8**

| **Target** | **Subanalysis** |  | **ΔTIS^∞^ Glands vs adjacent tissue** | **P value** | **ΔTIS^∞^ Stroma vs adjacent tissue** | **P value** | **ΔTIS^∞^ Glands vs stroma** | **P value** | **ΔTIS^∞^ Adjacent tissue vs healthy specimen** | **P value** |
| --- | --- | --- | --- | --- | --- | --- | --- | --- | --- | --- |
| **MMP3** | All endometriosis types |  | 6.38 | <0.001 | -2.55 | <0.001 | 8.93 | <0.001 | -0.54 | 0.348 |
|  | Subtypes | Peritoneal | 7.17 | <0.001 | -1.90 | <0.001 | 9.07 | <0.001 |  |  |
|  |  | Deep | 5.99 | <0.001 | -2.86 | <0.001 | 8.85 | <0.001 |  |  |
|  | Hormonal medication | Hormonal medication | 5.51 | <0.001 | -2.83 | <0.001 | 8.34 | <0.001 | -0.42 | 0.573 |
|  |  | No hormonal medication | 7.29 | <0.001 | -2.22 | <0.001 | 9.52 | <0.001 | -0.64 | 0.472 |

Supplementary table 2I. **Statistical analysis of total immunostaining score for MMP3**

| **Target** | **Subanalysis** |  | **ΔTIS^∞^ Glands vs adjacent tissue** | **P value** | **ΔTIS^∞^ Stroma vs adjacent tissue** | **P value** | **ΔTIS^∞^ Glands vs stroma** | **P value** | **ΔTIS^∞^ Adjacent tissue vs healthy specimen** | **P value** |
| --- | --- | --- | --- | --- | --- | --- | --- | --- | --- | --- |
| **MMP7** | All endometriosis types |  | 2.04 | <0.001 | 0.17 | 0.662 | 1.87 | <0.001 | 0.72 | 0.187 |
|  | Subtypes | Peritoneal | 2.11 | 0.002 | -0.71 | 0.279 | 2.82 | <0.001 |  |  |
|  |  | Deep | 2.00 | <0.001 | 0.62 | 0.189 | 1.38 | 0.004 |  |  |
|  | Hormonal medication | Hormonal medication | 1.82 | <0.001 | 0.50 | 0.364 | 1.32 | 0.018 | 0.28 | 0.694 |
|  |  | No hormonal medication | 2.26 | <0.001 | -0.15 | 0.784 | 2.41 | <0.001 | 1.43 | 0.091 |

Supplementary table 2J. **Statistical analysis of total immunostaining score for MMP7**

Supplementary table 2(A-J). **Statistical analysis of total immunostaining score (TIS) for all biomarkers. ^∞^ ΔTIS***: difference in Total Immunostaining Score, observed in immunohistochemical staining.*
